# Evaluating the Evidence: Scientometric Analysis of Highly Cited Neurofibromatosis 1 Publications

**DOI:** 10.7759/cureus.23466

**Published:** 2022-03-24

**Authors:** David R Hallan, Christopher Messner, Lekhaj C Daggubati, Surav Sakya, Sydnie Thomas, Elias Rizk

**Affiliations:** 1 Neurosurgery, Penn State Health Milton S. Hershey Medical Center, Hershey, USA; 2 Dermatology, Penn State College of Medicine, Hershey, USA; 3 Medicine, Penn State College of Medicine, Hershey, USA

**Keywords:** bibliometric, cited, evidence, grade, neurofibromatosis, nf1, scientometric

## Abstract

The study of Neurofibromatosis 1 (NF1) is progressing rapidly. This study aimed to identify historical trends in publications focusing on NF1, to find the top 100 most cited publications on this topic, and to evaluate their level of evidence. This study identifies historical trends in publication regarding NF1 with the aim of providing readers useful information about the areas of research being performed, an educational guide to facilitate novice researchers in conducting effective evidence-based medical research, and unique insight into developments and trends of NF 1 research. This study also evaluates the evidence of highly cited papers on NF1.

A search of all databases and journals accessible within Elsevier's Scopus was performed on June 27th, 2020, using combinations of the Boolean queries "Neurofibromatosis 1," "Von Recklinghausen," and "NF1," which yielded 13,599 documents. The top 100 most-cited papers were identified, analyzed, and evaluated for level of evidence. Evidence was assessed using the GRADE guidelines.

The top 100 most-cited articles span years 1963-2010 and are published in 50 different journals. The average number of citations per publication was 366.5 (range 189-1527). The most cited article is "Neurofibromatosis: Conference Statement" (Stumpf et al., 1988). In this study, the top 100 most-cited works in NF1 are identified, characterized, and analyzed. This study will serve as a historical point of reference for future research, a jumping point for those unfamiliar with the topic, and an educational foundation for future NF1 specialists and researchers.

## Introduction and background

Many medical specialties use bibliometrics to compile, publish, and review the most-cited works within their respective fields [1]. This is especially useful as the exponential rise in publications and resources makes it difficult for learners to process information efficiently. It likewise identifies and assesses the impact of publications, journals, and authors. Furthermore, bibliometrics takes a snapshot in time of objective metrics, which can highlight scientific progression, historical trends, and prolific individuals.

This study aimed to identify historical trends in publications focusing on Neurofibromatosis 1 (NF1), to find the top 100 most cited publications on this topic, and to evaluate their level of evidence. Our literature search revealed this had not previously been done. Reflecting not only on the trends of this research, but also their level of evidence, provides readers useful information about the areas of research being performed, provides an educational guide to facilitate novice researchers in performing effective evidence-based medical research, and provides unique insight into developments and trends of NF1 research.

## Review

Methods

A search of all databases and journals accessible within Elsevier's Scopus was performed on June 27th, 2020. Document search was performed using the Boolean query "[TITLE-ABS-KEY ( "Neurofibromatosis type 1" ) OR TITLE-ABS-KEY ( "Neurofibromatosis 1" ) OR TITLE-ABS-KEY ( "Von Recklinghausen's disease" ) OR TITLE-ABS-KEY ( "Von Recklinghausen" )]," without limitations on year or article type; and, which yielded 13,599 documents. Documents were ordered by the highest citations and screened for those papers whose primary focus was on NF1 or the NF1 gene, as well as the disease's complications, incidence, management, pathogenesis, treatment, and diagnostic evaluation. The top 100 most-cited papers from Elsevier's database were identified, and data were extracted. Data about these articles were collected and sorted. All references contained within the top 100 articles were obtained, sorted, and counted. Statistical analysis was performed using a combination of R-Studio and Bibliometrix [2]. The spectrographic analysis was performed using CRExplorer [3]. Two separate reviewers evaluated papers for the level of evidence using the GRADE system described by Guyatt et al. [4-6]. Any discrepancies in scoring were resolved by a third reviewer and discussion to reach a consensus. Papers that did not provide new evidence such as review papers and conference statements were excluded from this evaluation. Graphs and tables were drawn to illustrate the relationships between factors.

Results

The top 100 most-cited articles for NF1 span between 1963-2010 and are published in 50 journals. The average years from publication was 29.8, and the average number of citations per publication was 366.5, with an average of 12.3 citations per year. The total number of references contained within the top 100 articles was 3,852. Of the top 100 most-cited articles, the total number of citations ranges from 189-1527 (Table 1). The most cited article is "Neurofibromatosis: Conference Statement" by Stumpf et al. in 1988 from the Archives of Neurology with 1527 citations (Table 1) [7].

**Table 1:** The Top 100 Most Cited Articles for Neurofibromatosis 1

#	Title	Authors	Year	Source title	Cited by	Avg Citations per Year
1	Neurofibromatosis: Conference Statement	Stumpf DA, Alksne JF, Annegers JF, Brown SS, Conneally PM, Leppert MF, Miller JP, Moss ML, Pileggi AJ, Rapin I, Strohman RC, Swanson LW, Zimmerman A.	1988	Archives of Neurology	1527	47.7188
2	Type 1 neurofibromatosis gene: Identification of a large transcript disrupted in three NF1 patients	Wallace M.R., Marchuk D.A., Andersen L.B., Letcher R., Odeh H.M., Saulino A.M., Fountain J.W., Brereton A., Nicholson J., Mitchell A.L., Brownstein B.H., Collins F.S.	1990	Science	1102	36.7333
3	Malignant peripheral nerve sheath tumors. A clinicopathologic study of 120 cases	Ducatman B.S., Scheithauer B.W., Piepgras D.G., Reiman H.M., Ilstrup D.M.	1986	Cancer	1034	30.4118
4	The diagnostic evaluation and multidisciplinary management of neurofibromatosis 1 and neurofibromatosis 2	Gutmann D.H., Aylsworth A., Carey J.C., Korf B., Marks J., Pyeritz R.E., Rubenstein A., Viskochil D.	1997	Journal of the American Medical Association	1017	44.2174
5	Von Recklinghausen Neurofibromatosis	Riccardi V.M.	1981	New England Journal of Medicine	912	23.3846
6	A major segment of the neurofibromatosis type 1 gene: cDNA sequence, genomic structure, and point mutations	Cawthon R.M., Weiss R., Xu G., Viskochil D., Culver M., Stevens J., Robertson M., Dunn D., Gesteland R., O'Connell P., White R.	1990	Cell	887	29.5667
7	The neurofibromatosis type 1 gene encodes a protein related to GAP	Xu G., O'Connell P., Viskochil D., Cawthon R., Robertson M., Culver M., Dunn D., Stevens J., Gesteland R., White R., Weiss R.	1990	Cell	833	27.7667
8	Deletions and a translocation interrupt a cloned gene at the neurofibromatosis type 1 locus	Viskochil D., Buchberg A.M., Xu G., Cawthon R.M., Stevens J., Wolff R.K., Culver M., Carey J.C., Copeland N.G., Jenkins N.A., White R., O'Connell P.	1990	Cell	813	27.1
9	Malignant peripheral nerve sheath tumours in neurofibromatosis	Evans D.G.R., Baser M.E., McGaughran J., Sharif S., Howard E., Moran A.	2002	Journal of Medical Genetics	699	38.8333
10	The GAP-related domain of the neurofibromatosis type 1 gene product interacts with ras p21	Martin G.A., Viskoohil D., Bollag G., McCabe P.C., Crosier W.J., Haubruck H., Conroy L., Clark R., O'Connell P., Cawthon R.M., Innis M.A., McCormick F.	1990	Cell	677	22.5667
11	Tumour predisposition in mice heterozygous for a targeted mutation in Nf1	Jacks T., Shih T.S., Schmitt E.M., Bronson R.T., Bernards A., Weinberg R.A.	1994	Nature Genetics	600	23.0769
12	The NF1 locus encodes a protein functionally related to mammalian GAP and yeast IRA proteins	Ballester R., Marchuk D., Boguski M., Saulino A., Letcher R., Wigler M., Collins F.	1990	Cell	598	19.9333
13	Aberrant regulation of ras proteins in malignant tumour cells from type 1 neurofibromatosis patients	Basu T.N., Gutmann D.H., Fletcher J.A., Glover T.W., Collins F.S., Downward J.	1992	Nature	525	18.75
14	The catalytic domain of the neurofibromatosis type 1 gene product stimulates ras GTPase and complements ira mutants of S. cerevisiae	Xu G., Lin B., Tanaka K., Dunn D., Wood D., Gesteland R., White R., Weiss R., Tamanoi F.	1990	Cell	513	17.1
15	Von recklinghausen neurofibromatosis: A clinical and population study in south-east Wales	Huson S.M., Harper P.S., Compston D.A.S.	1988	Brain	509	15.9063
16	Guidelines for the diagnosis and management of individuals with neurofibromatosis	Ferner R.E., Huson S.M., Thomas N., Moss C., Willshaw H., Evans D.G., Upadhyaya M., Towers R., Gleeson M., Steiger C., Kirby A.	2007	Journal of Medical Genetics	500	38.4615
17	Gene for von Recklinghausen neurofibromatosis is in the pericentromeric region of chromosome 17	Barker D., Wright E., Nguyen K., Cannon L., Fain P., Goldgar D., Bishop D.T., Carey J., Baty B., Kivlin J., Willard H., Waye J.S., Greig G., Leinwand L., Nakamura Y., O'Connell P., Leppert M., Lalouel J.-M., White R., Skolnick M.	1987	Science	493	14.9394
18	Abnormal regulation of mammalian p21ras contributes to malignant tumor growth in von Recklinghausen (type 1) neurofibromatosis	DeClue J.E., Papageorge A.G., Fletcher J.A., Diehl S.R., Ratner N., Vass W.C., Lowy D.R.	1992	Cell	485	17.3214
19	Targeted disruption of the neurofibromatosis type-1 gene leads to developmental abnormalities in heart and various neural crest-derived tissues	Brannan C.I., Perkins A.S., Vogel K.S., Ratner N., Nordlund M.L., Reid S.W., Buchberg A.M., Jenkins N.A., Parada L.F., Copeland N.G.	1994	Genes and Development	484	18.6154
20	Long-Term Follow-up of von Recklinghausen Neurofibromatosis	Sørensen S.A., Mulvihill J.J., Nielsen A.	1986	New England Journal of Medicine	443	13.0294
21	Loss of NF1 results in activation of the Ras signaling pathway and leads to aberrant growth in haematopoietic cells	Bollag G., Clapp D.W., Shih S., Adler F., Zhang Y.Y., Thompson P., Lange B.J., Freedman M.H., McCormick F., Jacks T., Shannon K.	1996	Nature Genetics	429	17.875
22	The NF1 tumor suppressor critically regulates TSC2 and mTOR	Johannessen C.M., Reczek E.E., James M.F., Brems H., Legius E., Cichowski K.	2005	Proceedings of the National Academy of Sciences of the United States of America	423	28.2
23	Neurofibromas in NF1: Schwann cell origin and role of tumor environment	Zhu Y., Ghosh P., Charnay P., Burns D.K., Parada L.F.	2002	Science	422	23.4444
24	Mechanism for the learning deficits in a mouse model of neurofibromatosis type 1	Costa R.M., Federov N.B., Kogan J.H., Murphy G.G., Stern J., Ohno M., Kucherlapati R., Jacks T., Silva A.J.	2002	Nature	408	22.6667
25	Natural history of optic pathway tumors in children with neurofibromatosis type 1: A longitudinal study	Listernick R., Charrow J., Greenwald M., Mets M.	1994	Journal of Pediatrics	391	15.0385
26	International consensus statement on malignant peripheral nerve sheath tumors in neurofibromatosis	Ferner R.E., Gutmann D.H.	2002	Cancer Research	379	21.0556
27	Ablation of NF1 function in neurons induces abnormal development of cerebral cortex and reactive gliosis in the brain	Zhu Y., Romero M.I., Ghosh P., Ye Z., Charnay P., Rushing E.J., Marth J.D., Parada L.F.	2001	Genes and Development	376	19.7895
28	A genetic study of von Recklinghausen neurofibromatosis in south east Wales. I Prevalence, fitness, mutation rate, and effect of parental transmission on severity	Huson S.M., Compston D.A.S., Clark P., Harper P.S.	1989	Journal of Medical Genetics	368	11.871
29	Epidemiology of neurofibromatosis type 1	Friedman J.M.	1999	American Journal of Medical Genetics - Seminars in Medical Genetics	360	17.1429
30	Optic pathway gliomas in neurofibromatosis-1: Controversies and recommendations	Listernick R., Ferner R.E., Liu G.T., Gutmann D.H.	2007	Annals of Neurology	354	27.2308
31	Neurofibromatosis type 1 revisited	Williams V.C., Lucas J., Babcock M.A., Gutmann D.H., Bruce B., Maria B.L.	2009	Pediatrics	353	32.0909
32	Early inactivation of p53 tumor suppressor gene cooperating with NF1 loss induces malignant astrocytoma	Zhu Y., Guignard F., Zhao D., Liu L., Burns D.K., Mason R.P., Messing A., Parada L.F.	2005	Cancer Cell	351	23.4
33	Exhaustive mutation analysis of the NF1 gene allows identification of 95% of mutations and reveals a high frequency of unusual splicing defects	Messiaen L.M., Callens T., Mortier G., Beysen D., Vandenbroucke I., Van Roy N., Speleman F., De Paepe A.	2000	Human Mutation	347	17.35
34	Optic pathway gliomas in children with neurofibromatosis 1: Consensus statement from the NF1 optic pathway glioma task force	Listernick R., Louis D.N., Packer R.J., Gutmann D.H.	1997	Annals of Neurology	343	14.913
35	A de novo Alu insertion results in neurofibromatosis type 1	Wallace M.R., Andersen L.B., Saulino A.M., Gregory P.E., Glover T.W., Collins F.S.	1991	Nature	342	11.7931
36	The nature and frequency of cognitive deficits in children with neurofibromatosis type 1	Hyman S.L., Shores A., North K.N.	2005	Neurology	339	22.6
37	Loss of the normal NF1 allele from the bone marrow of children with type 1 neurofibromatosis and malignant myeloid disorders	Shannon K.M., O'connell P., Martin G.A., Paderanga D., Olson K., Dinndorf P., Mccormick F.	1994	New England Journal of Medicine	339	13.0385
38	Neurofibromatosis 1 (Recklinghausen disease) and neurofibromatosis 2 (bilateral acoustic neurofibromatosis): An update	Mulvihill J.J., Parry D.M., Sherman J.L., Pikus A., Kaiser-Kupfer M.I., Eldridge R.	1990	Annals of Internal Medicine	334	11.1333
39	Mouse models of tumor development in neurofibromatosis type 1	Cichowski K., Shih T.S., Schmitt E., Santiago S., Reilly K., McLaughlin M.E., Bronson R.T., Jacks T.	1999	Science	330	15.7143
40	Chromosome 17p deletions and p53 gene mutations associated with the formation of malignant neurofibrosarcomas in von Recklinghausen neurofibromatosis	Menon A.G., Anderson K.M., Riccardi V.M., Chung R.Y., Whaley J.M., Yandell D.W., Farmer G.E., Freiman R.N., Lee J.K., Li F.P., Barker D.F., Ledbetter D.H., Kleider A., Martuza R.L., Gusella J.F., Seizinger B.R.	1990	Proceedings of the National Academy of Sciences of the United States of America	325	10.8333
41	Somatic deletion of the neurofibromatosis type 1 gene in a neurofibrosarcoma supports a tumour suppressor gene hypothesis	Legius E., Marchuk D.A., Collins F.S., Glover T.W.	1993	Nature Genetics	324	12
42	cDNA cloning of the type 1 neurofibromatosis gene: Complete sequence of the NF1 gene product	Marchuk D.A., Saulino A.M., Tavakkol R., Swaroop M., Wallace M.R., Andersen L.B., Mitchell A.L., Gutmann D.H., Boguski M., Collins F.S.	1991	Genomics	323	11.1379
43	Gastrointestinal stromal tumors in patients with neurofibromatosis 1: A clinicopathologic and molecular genetic study of 45 cases	Miettinen M., Fetsch J.F., Sobin L.H., Lasota J.	2006	American Journal of Surgical Pathology	315	22.5
44	Mortality in neurofibromatosis 1: An analysis using U.S. death certificates	Rasmussen S.A., Yang Q., Friedman J.M.	2001	American Journal of Human Genetics	314	16.5263
45	Use of the National Institutes of Health criteria for diagnosis of neurofibromatosis 1 in children	DeBella K., Szudek J., Friedman J.M.	2000	Pediatrics	310	15.5
46	Type 1 neurofibromatosis: A descriptive analysis of the disorder in 1,728 patients	Friedman J.M., Birch P.H.	1997	American Journal of Medical Genetics	299	13
47	The HMG-CoA reductase inhibitor lovastatin reverses the learning and attention deficits in a mouse model of Neurofibromatosis Type 1	Li W., Cui Y., Kushner S.A., Brown R.A.M., Jentsch J.D., Frankland P.W., Cannon T.D., Silva A.J.	2005	Current Biology	291	19.4
48	Neurofibromatosis 1 and neurofibromatosis 2: a twenty first century perspective	Ferner R.E.	2007	Lancet Neurology	288	22.1538
49	Somatic mutations in the neurofibromatosis 1 gene in human tumors	Li Y., Bollag G., Clark R., Stevens J., Conroy L., Fults D., Ward K., Friedman E., Samowitz W., Robertson M., Bradley P., McCormick F., White R., Cawthon R.	1992	Cell	288	10.2857
50	Malignant peripheral nerve sheath tumor: Analysis of treatment outcome	Wong W.W., Hirose T., Scheithauer B.W., Schild S.E., Gunderson L.L.	1998	International Journal of Radiation Oncology Biology Physics	286	13
51	Von Recklinghausen's disease: a clinicopathological study.	Brasfield R.D., Das Gupta T.K.	1972	Annals of surgery	286	5.95833
52	An analysis of variation in expression of neurofibromatosis (NF) type 1 (NF1): Evidence for modifying genes	Easton D.F., Ponder M.A., Huson S.M., Ponder B.A.J.	1993	American Journal of Human Genetics	283	10.4815
53	Peripheral nerve tumors with rhabdomyosarcomatous differentiation (malignant “triton” tumors)	Woodruff J.M., Chernik N.L., Smith M.C., Millett W.B., Foote F.W., JR.	1973	Cancer	276	5.87234
54	Sarcomas of the peripheral nerves and somatic soft tissues associated with multiple neurofibromatosis (von Recklinghausen's disease)	D'Agostino A.N., Soule E.H., Miller R.H.	1963	Cancer	276	4.84211
55	Neurofibromin Regulation of ERK Signaling Modulates GABA Release and Learning	Cui Y., Costa R.M., Murphy G.G., Elgersma Y., Zhu Y., Gutmann D.H., Parada L.F., Mody I., Silva A.J.	2008	Cell	269	22.4167
56	Nf1;Trp53 mutant mice develop glioblastoma with evidence of strain-specific effects	Reilly K.M., Loisel D.A., Bronson R.T., McLaughlin M.E., Jacks T.	2000	Nature Genetics	265	13.25
57	A mouse model for the learning and memory deficits associated with neurofibromatosis type I	Silva A.J., Frankland P.W., Marowitz Z., Friedman E., Lazlo G., Cioffi D., Jacks T., Bourtchuladze R.	1997	Nature Genetics	265	11.5217
58	NF1 gene and neurofibromatosis 1	Rasmussen S.A., Friedman J.M.	2000	American Journal of Epidemiology	261	13.05
59	Molecular genetics of neurofibromatosis type 1 (NF1)	Shen M.H., Harper P.S., Upadhyaya M.	1996	Journal of Medical Genetics	261	10.875
60	Malignant peripheral nerve sheath tumors of the buttock and lower extremity. A study of 43 cases	Hruban R.H., Shiu M.H., Senie R.T., Woodruff J.M.	1990	Cancer	261	8.7
61	Mouse tumor model for neurofibromatosis type 1	Vogel K.S., Klesse L.J., Velasco-Miguel S., Meyers K., Rushing E.J., Parada L.F.	1999	Science	257	12.2381
62	Plexiform neurofibromas	Korf B.R.	1999	American Journal of Medical Genetics - Seminars in Medical Genetics	257	12.2381
63	Malignant peripheral nerve sheath tumors. A clinicopathologic study of 28 cases	Wanebo J.E., Malik J.M., Vandenberg S.R., Wanebo H.J., Driesen N., Persing J.A.	1993	Cancer	254	9.40741
64	Von Recklinghausen's disease and pheochromocytomas	Walther M.M., Herring J., Enquist E., Keiser H.R., Linehan W.M.	1999	Journal of Urology	252	12
65	Genetic linkage of von Recklinghausen neurofibromatosis to the nerve growth factor receptor gene	Seizinger B.R., Rouleau G.A., Ozelius L.J., Lane A.H., Faryniarz A.G., Chao M.V., Huson S., Korf B.R., Parry D.M., Pericak-Vance M.A., Collins F.S., Hobbs W.J., Falcone B.G., Iannazzi J.A., Roy J.C., St George-Hyslop P.H., Tanzi R.E., Bothwell M.A., Upadhyaya M., Harper P., Goldstein A.E., Hoover D.L., Bader J.L., Spence M.A., Mulvihill J.J., Aylsworth A.S., Vance J.M., Rossenwasser G.O.D., Gaskell P.C., Roses A.D., Martuza R.L., Breakefield X.O., Gusella J.F.	1987	Cell	252	7.63636
66	The clinical and diagnostic implications of mosaicism in the neurofibromatoses	Ruggieri M., Huson S.M.	2001	Neurology	249	13.1053
67	Differential regulation of rasGAP and neurofibromatosis gene product activities	Bollag G., McCormick F.	1991	Nature	246	8.48276
68	Nf1 deficiency causes Ras-mediated granulocyte/macrophage colony stimulating factor hypersensitivity and chronic myeloid leukaemia	Largaespada D.A., Brannan C.I., Jenkins N.A., Copeland N.G.	1996	Nature Genetics	244	10.1667
69	Mutations affecting mRNA splicing are the most common molecular defects in patients with neurofibromatosis type 1	Ars E., Serra E., García J., Kruyer H., Gaona A., Lázaro C., Estivill X.	2000	Human Molecular Genetics	243	12.15
70	Neurofibromatosis type 1	Boyd K.P., Korf B.R., Theos A.	2009	Journal of the American Academy of Dermatology	240	21.8182
71	Nf1-Dependent Tumors Require a Microenvironment Containing Nf1+/-- and c-kit-Dependent Bone Marrow	Yang F.-C., Ingram D.A., Chen S., Zhu Y., Yuan J., Li X., Yang X., Knowles S., Horn W., Li Y., Zhang S., Yang Y., Vakili S.T., Yu M., Burns D., Robertson K., Hutchins G., Parada L.F., Clapp D.W.	2008	Cell	240	20
72	Clinical and genetic aspects of neurofibromatosis 1	Jett K., Friedman J.M.	2010	Genetics in Medicine	237	23.7
73	Neurofibromatosis: Clinical heterogeneity	Riccardi V.M.	1982	Current Problems in Cancer	237	6.23684
74	Cardiovascular disease in neurofibromatosis 1: Report of the NF1 Cardiovascular Task Force	Friedman J.M., Arbiter J., Epstein J.A., Gutmann D.H., Huot S.J., Lin A.E., McManus B., Korf B.R.	2002	Genetics in Medicine	236	13.1111
75	von Recklinghausen Neurofibromatosis: II. Incidence of Optic Gliomata	Lewis R.A., Gerson L.P., Axelson K.A., Riccardi V.M., Whitford R.P.	1984	Ophthalmology	235	6.52778
76	The protein product of the neurofibromatosis type 1 gene is expressed at highest abundance in neurons, Schwann cells, and oligodendrocytes	Daston M.M., Scrable H., Nordlund M., Sturbaum A.K., Nissen L.M., Ratner N.	1992	Neuron	234	8.35714
77	Optic gliomas in children with neurofibromatosis type 1	Listernick R., Charrow J., Greenwald M.J., Esterly N.B.	1989	The Journal of Pediatrics	234	7.54839
78	Malignancy in neurofibromatosis type 1	Korf B.R.	2000	Oncologist	224	11.2
79	The Ras/Raf/ERK signalling pathway drives Schwann cell dedifferentiation	Harrisingh M.C., Perez-Nadales E., Parkinson D.B., Malcolm D.S., Mudge A.W., Lloyd A.C.	2004	EMBO Journal	223	13.9375
80	Malignant peripheral nerve sheath tumors: Prognostic factors and survival in a series of patients treated at a single institution	Anghileri M., Miceli R., Fiore M., Mariani L., Ferrari A., Mussi C., Lozza L., Collini P., Olmi P., Casali P.G., Pilotti S., Gronchi A.	2006	Cancer	221	15.7857
81	Neurofibromatosis and childhood leukemia	Bader J.L., Miller R.W.	1978	The Journal of Pediatrics	220	5.2381
82	Minor lesion mutational spectrum of the entire NF1 gene does not explain its high mutability but points to a functional domain upstream of the GAP- related domain	Fahsold R., Hoffmeyer S., Mischung C., Gille C., Ehlers C., Kücükceylan N., Abdel-Nour M., Gewies A., Peters H., Kaufmann D., Buske A., Tinschert S., Nürnberg P.	2000	American Journal of Human Genetics	217	10.85
83	Homozygous inactivation of the NF1 gene in bone marrow cells from children with neurofibromatosis type 1 and malignant myeloid disorders	Side L., Taylor B., Cayouette M., Conner E., Thompson P., Luce M., Shannon K.	1997	New England Journal of Medicine	217	9.43478
84	Astrocyte-specific inactivation of the neurofibromatosis 1 gene (NF1) is insufficient for astrocytoma formation	Bajenaru M.L., Zhu Y., Hedrick N.M., Donahoe J., Parada L.F., Gutmann D.H.	2002	Molecular and Cellular Biology	216	12
85	Malignancy in neurofibromatosis.	Hope D.G., Mulvihill J.J.	1981	Advances in neurology	213	5.46154
86	Prevalence of neurofibromatosis 1 in German children at elementary school enrollment	Lammert M., Friedman J.M., Kluwe L., Mautner V.F.	2005	Archives of Dermatology	210	14
87	Gastrointestinal manifestations of type 1 neurofibromatosis (von Recklinghausen's disease)	FLULLER C.E., WILLIAMS G.T.	1991	Histopathology	208	7.17241
88	The vascular lesions of neurofibromatosis	Salyer W.R., Salyer D.C.	1974	Angiology	208	4.52174
89	Identification of the neurofibromatosis type 1 gene product	Gutmann D.H., Wood D.L., Collins F.S.	1991	Proceedings of the National Academy of Sciences of the United States of America	207	7.13793
90	Second primary tumors in neurofibromatosis 1 patients treated for optic glioma: Substantial risks after radiotherapy	Sharif S., Ferner R., Birch J.M., Gillespie J.E., Gattamaneni H.R., Baser M.E., Evans D.G.R.	2006	Journal of Clinical Oncology	206	14.7143
91	Cognitive function and academic performance in neurofibromatosis 1: Consensus statement from the NF1 cognitive disorders task force	North K.N., Riccardi V., Samango-Sprouse C., Ferner R., Moore B., Legius E., Ratner N., Denckla M.B.	1997	Neurology	206	8.95652
92	Neurofibromatosis type 1: Pathologic substrate of high-signal-intensity foci in the brain	DiPaolo D.P., Zimmerman R.A., Rorke L.B., Zackai E.H., Bilaniuk L.T., Yachnis A.T.	1995	Radiology	206	8.24
93	Mechanisms in the pathogenesis of malignant tumours in neurofibromatosis type 1	Brems H., Beert E., de Ravel T., Legius E.	2009	The Lancet Oncology	204	18.5455
94	Proteomic analysis reveals hyperactivation of the mammalian target of rapamycin pathway in neurofibromatosis 1-associated human and mouse brain tumors	Dasgupta B., Yi Y., Chen D.Y., Weber J.D., Gutmann D.H.	2005	Cancer Research	204	13.6
95	An absence of cutaneous neurofibromas associated with a 3-bp inframe deletion in exon 17 of the NF1 gene (c.2970-2972 delAAT): Evidence of a clinically significant NF1 genotype-phenotype correlation	Upadhyaya M., Huson S.M., Davies M., Thomas N., Chuzhanova N., Giovannini S., Evans D.G., Howard E., Kerr B., Griffiths S., Consoli C., Side L., Adams D., Pierpont M., Hachen R., Barnicoat A., Li H., Wallace P., Van Biervliet J.P., Stevenson D., Viskochil D., Baralle D., Haan E., Riccardi V., Turnpenny P., Lazaro C., Messiaen L.	2007	American Journal of Human Genetics	203	15.6154
96	Repair of the lower and middle parts of the face by composite tissue allotransplantation in a patient with massive plexiform neurofibroma: a 1-year follow-up study	Lantieri L., Meningaud J.-P., Grimbert P., Bellivier F., Lefaucheur J.-P., Ortonne N., Benjoar M.-D., Lang P., Wolkenstein P.	2008	The Lancet	202	16.8333
97	Pediatric malignant peripheral nerve sheath tumor: The Italian and German Soft Tissue Sarcoma Cooperative Group	Carli M., Ferrari A., Mattke A., Zanetti I., Casanova M., Bisogno G., Cecchetto G., Alaggio R., De Sio L., Koscielniak E., Sotti G., Treuner J.	2005	Journal of Clinical Oncology	199	13.2667
98	Optic Nerve Glioma in Mice Requires Astrocyte Nf1 Gene Inactivation and Nf1 Brain Heterozygosity	Bajenaru M.L., Hernandez M.R., Perry A., Zhu Y., Parada L.F., Garbow J.R., Gutmann D.H.	2003	Cancer Research	199	11.7059
99	Neurofibromatosis and childhood leukaemia/lymphoma: A population-based UKCCSG study	Stiller C.A., Chessells J.M., Fitchett M.	1994	British Journal of Cancer	195	7.5
100	NF1-associated gastrointestinal stromal tumors have unique clinical, phenotypic, and genotypic characteristics	Andersson J., Sihto H., Meis-Kindblom J.M., Joensuu H., Nupponen N., Kindblom L.-G.	2005	American Journal of Surgical Pathology	189	12.6

**Figure 1:**
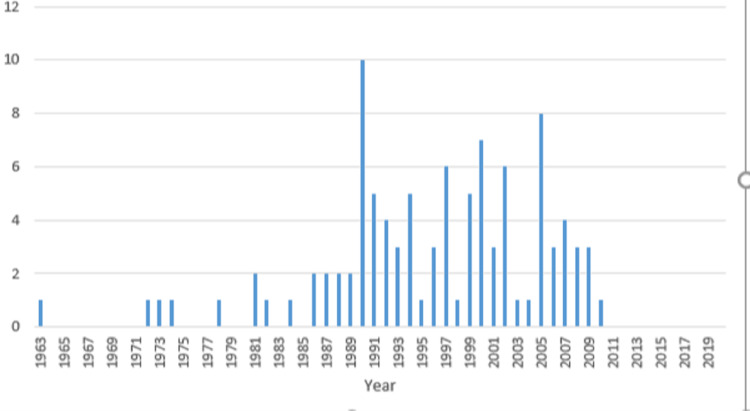
Publications, by year, within the top 100 most-cited articles. *Journals with only one publication were excluded from the figure but are as follows: Advances in Neurology, American Journal of Epidemiology, American Journal of Medical Genetics, Angiology, Annals of Internal Medicine, Annals of Surgery, Archives of Dermatology, Archives of Neurology, Brain, British Journal of Cancer, Cancer Cell, Current Biology, Current Problems In Cancer, Embo Journal, Genomics, Histopathology, Human Molecular Genetics, Human Mutation, International Journal of Radiation Oncology Biology Physics, Journal of Pediatrics, Journal of the American Academy of Dermatology, Journal of the American Medical Association, Journal of Urology, Lancet Neurology, Molecular, and Cellular Biology, Neuron, Oncologist, Ophthalmology, Radiology, The Lancet, The Lancet Oncology

The mode of publication year was 1990, with ten publications. The latest year of publication included in the Top 100 is 2010 with a single publication (Figure 1). The top 100 most-cited publications consisted of 82 original articles, 17 review articles, and one conference paper.

Only 44 articles had funding sponsors. The most common funding sponsor was the National Institutes of Health (9), followed by the National Cancer Institute (5), the American Cancer Society (3), Merck (3), the National Institute of Neurological Disorders and Stroke (2), and the United States Department of Defense (2).

**Figure 2:**
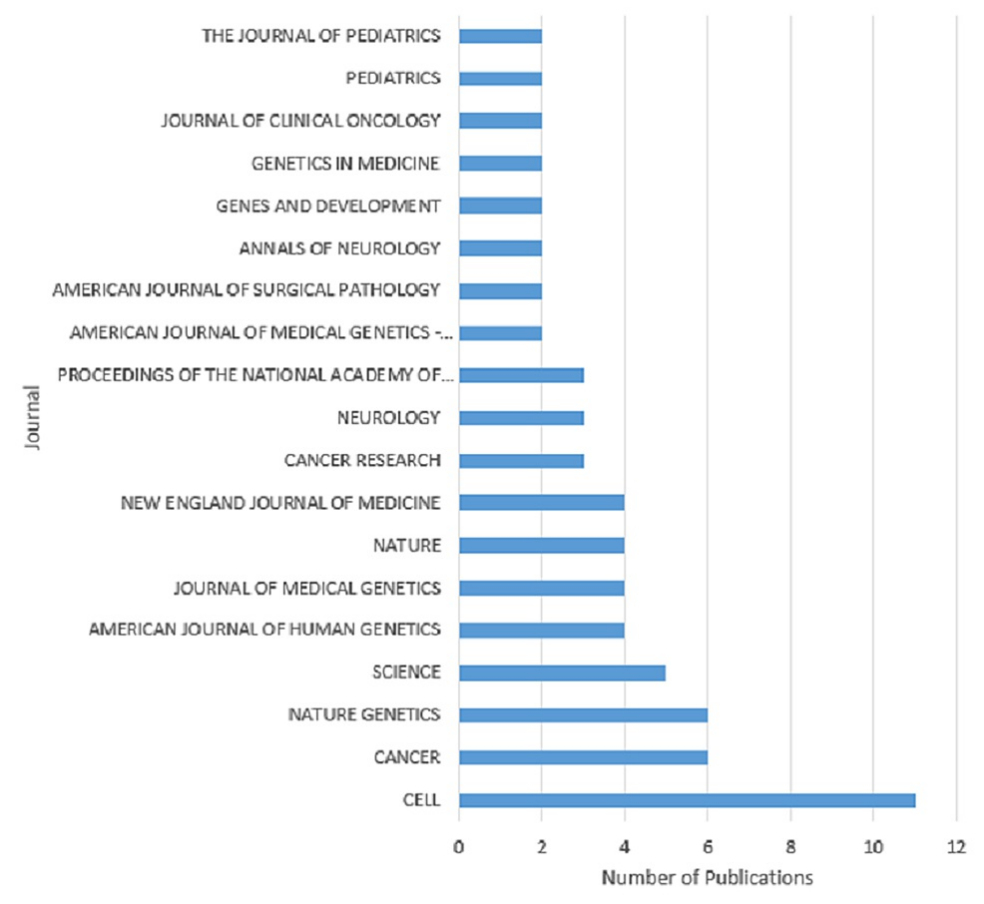
Journals in which the 100 most-cited articles were most frequently published *Journals with only one publication were excluded from the figure but are as follows: Advances in Neurology, American Journal of Epidemiology, American Journal of Medical Genetics, Angiology, Annals of Internal Medicine, Annals of Surgery, Archives of Dermatology, Archives of Neurology, Brain, British Journal of Cancer, Cancer Cell, Current Biology, Current Problems In Cancer, Embo Journal, Genomics, Histopathology, Human Molecular Genetics, Human Mutation, International Journal of Radiation Oncology Biology Physics, Journal of Pediatrics, Journal of the American Academy of Dermatology, Journal of the American Medical Association, Journal of Urology, Lancet Neurology, Molecular, and Cellular Biology, Neuron, Oncologist, Ophthalmology, Radiology, The Lancet, The Lancet Oncology

The top five journals these publications most frequently appeared in are (a) Cell with 11 publications, (b) Cancer with six, (c) Nature Genetics with six, (d) Science with five, and (e) The American Journal of Human Genetics, The Journal of Medical Genetics, Nature, and The New England Journal of Medicine with four publications each (Figure 2).

The author with the most publications (Table 2) and most local citations (Table 3) within the top 100 most cited was D.H. Gutmann, with 13 articles in the Top 100. An author's H-index did not correlate with the highest citation count or the number of publications within the top 100 (Table 2).

**Table 2:** Top 20 authors in top 100 most cited

Author	# Papers within Top 100	h_index	TC within Top 100	Starting Year Within Top 100
GUTMANN DH	13	87	4625	1991
PARADA LF	9	91	2814	1994
FRIEDMAN JM	8	69	2227	1997
COLLINS FS	7	176	3075	1987
ZHU Y	7	23	2073	2001
O'CONNELL P	6	61	4042	1987
WHITE R	6	84	3827	1987
JACKS T	6	132	2297	1994
HUSON SM	6	48	2112	1988
KORF BR	5	55	1209	1987
MCCORMICK F	5	113	1979	1990
VISKOCHIL D	5	49	3753	1990
BOLLAG GE	4	58	1640	1990
FERNER RE	4	36	1521	2002
LEGIUS E	4	60	1157	1993
LISTERNICK R	4	26	1322	1989
MULVIHILL JJ	4	59	1242	1981
RATNER N	4	51	1409	1992
RICCARDI VM	4	46	1709	1981
SILVA AJ	4	80	1233	1997

**Table 3:** Top 20 authors that were cited most by the top 100 publications

Authors	Citations
GUTMANN D H	118
RICCARDI V M	117
HUSON S M	79
FRIEDMAN J M	73
VISKOCHIL D	73
UPADHYAYA M	61
COLLINS F S	50
JACKS T	50
WALLACE M R	47
XU G	47
MARCHUK D A	46
LISTERNICK R	45
RATNER N	43
HARPER P S	37
O CONNELL P	37
LEGIUS E	36
MULVIHILL J J	34
CAWTHON R M	33
MAUTNER V F	33
CAREY J C	32

Overall, there were 470 authors, with 655 total author appearances. Seven of the publications were from single authors, but most publications averaged 4.7 authors per document.

**Figure 3:**
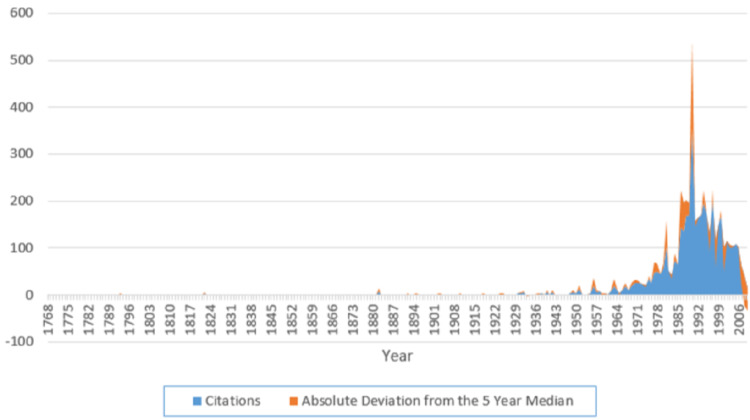
A spectrographic analysis of the 100 most-cited articles' 3,852 references, with references going as far back as the year 1768

The historical roots of NF1 research were identified using spectrographic analysis according to the method of Marx et al. 2014 [8]. The largest peak occurs in the year 1990, which is indicative of the year when NF1 research took its firmest foothold (Figure 3).

Spectrographic analysis reveals quantitatively which historical papers are of particular interest in this specific research topic. This mode of analysis combats "obliteration by incorporation," where novel ideas are "rapidly absorbed into the body of scientific knowledge and their origins thus quickly forgotten due to familiarity," and "palimpsestic syndrome," where an "idea is covered by ascribing it to a more recent author who cites the original work” [8].

**Figure 4:**
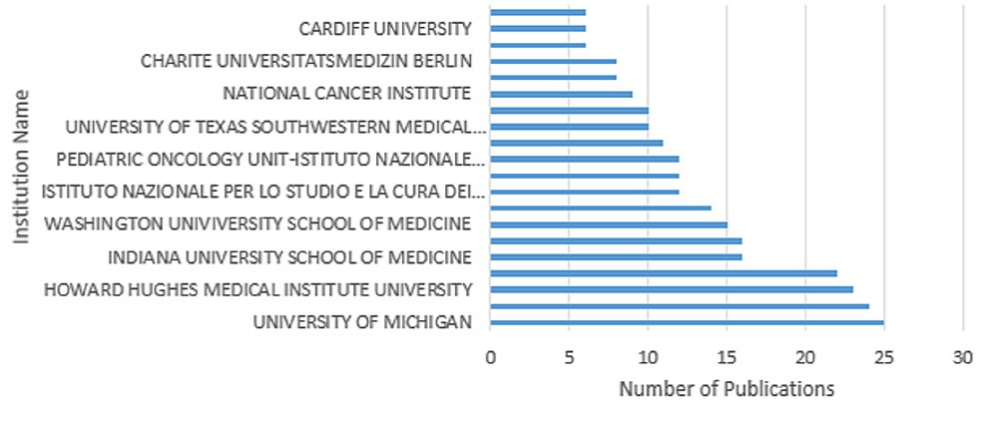
The top affiliations associated with the top 100 most-cited articles

The institution affiliated with the most publications in the top 100 was the University of Michigan with 25 publications (Figure 4). The University of California was associated with 24 publications, Howard Hughes Medical Institute University had 23, Harvard Medical School had 22, Indiana University School of Medicine and University of Utah Medical Center had 16, Washington University School of Medicine had 15, Massachusetts General Hospital had 14, Istituto Nazionale Per Lo Studio E La Cura Del Tumori, Olgahospital, and the Pediatric Oncology Unit-Istituto Nazionale Tumori had 12, the University of British Columbia had 11, University of Texas Southwestern Medical Center and the University of Wales College of Medicine had 10, the National Cancer Institute had nine, Ghent University Hospital and Charité Universitätsmedizin Berlin had eight, and Baylor College of Medicine, Cardiff University, and Children's Hospital of Philadelphia each had six (Figure 4).

**Figure 5:**
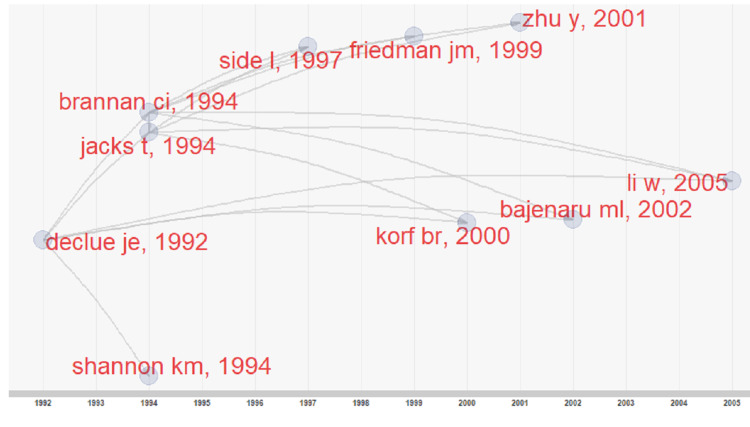
The top 100 most-cited articles' historical direct citation networks

Table 3 lists the authors that the top 100 most-cited articles cited most in their references. These authors all appear as authors of the top 100 most-cited articles. Table 4 lists the most cited documents by the top 100 most-cited articles. The most locally cited document was "Deletions and a Translocation Interrupt a Cloned Gene at the Neurofibromatosis Type 1 Locus" by Viskochil et al. in 1990 with 26 local citations and 813 global citations (Table 1 and Figure 5).

**Table 4:** Top 20 most cited documents by top 100 most cited articles

Document	Year	Local.Citations	Global.Citations
VISKOCHIL D, 1990, CELL	1990	26	813
WALLACE MR, 1990,	1990	23	1102
CAWTHON RM, 1990, CELL	1990	23	887
XU G, 1990, CELL	1990	21	833
RICCARDI VM, 1981, NEW ENGL J MED	1981	17	912
MARTIN GA, 1990, CELL	1990	15	677
GUTMANN DH, 1997, J AM MED ASSOC	1997	12	1017
XU G, 1990, CELL-a	1990	11	513
BALLESTER R, 1990, CELL	1990	10	598
BRANNAN CI, 1994, GENES DEV	1994	10	484
BADER JL, 1978, J PEDIATR	1978	10	220
DECLUE JE, 1992, CELL	1992	9	485
FRIEDMAN JM, 1997, AM J MED GENET	1997	9	299
HOPE DG, 1981, ADV NEUROL	1981	9	213
D'AGOSTINO AN, 1963, CANCER	1963	8	276
STUMPF DA, 1988, ARCH NEUROL	1988	7	1527
HUSON SM, 1988, BRAIN	1988	7	509
LEGIUS E, 1993, NAT GENET	1993	7	324
MARCHUK DA, 1991, GENOMICS	1991	7	323
EASTON DF, 1993, AM J HUM GENET	1993	7	283

The geographic distribution of corresponding authors with NF1 publications was made up of 59% publications from the US, 13% from the UK, 5% from Canada, 3% from Italy, 2% from Australia, Belgium, and Germany each, and 1% from Denmark, France, and Sweden (Figure 5). The top eight institutions contributing to the top 100 NF1 publications were all from the US, with the University of Michigan, University of California, Howard Hughes Medical Institute University, and Harvard Medical School, each being involved with more than 20 articles (Figure 4).

**Figure 6:**
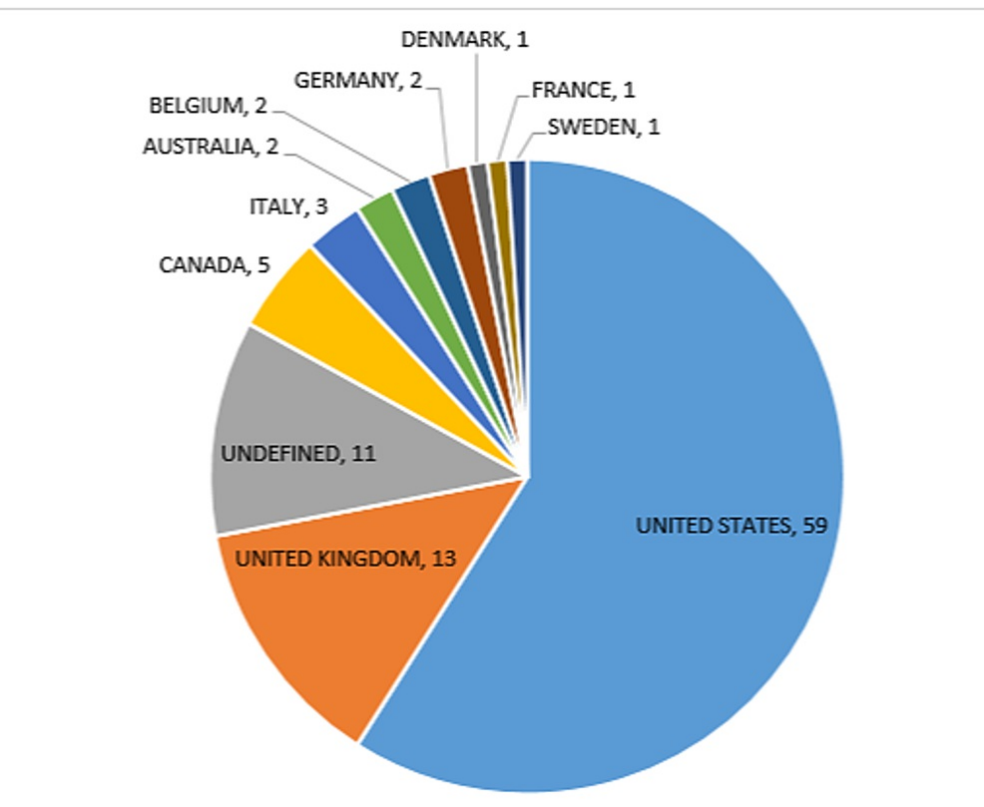
The country of origin for the top 100 most-cited articles, based on the country of the corresponding author

**Figure 7:**
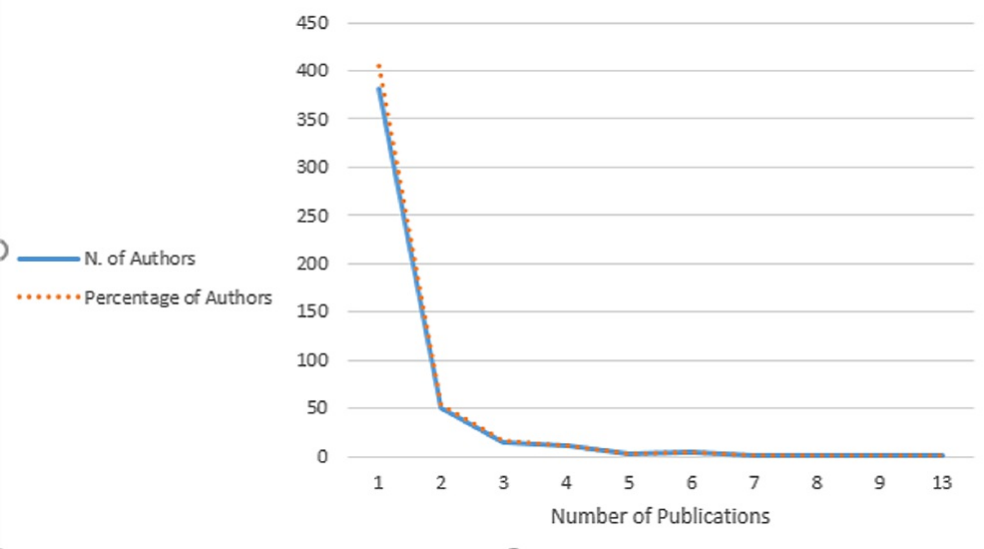
The distribution of publications by the author as a representation of Lotka's law

The distribution of publications by author (Figure 6) (Lotka's Law) [9] shows that 381 authors published one paper, and 51 authors published two papers within the top 100 most-cited. Another 38 authors published more than three papers, with the most with 13 articles. The historical origins of NF1 research were also traced using a direct citation network (Figure 7) to compare to the spectrographic analysis seen in Figure 3. Four distinct groupings were branching from Declue Je et al.'s paper in 1992.

**Figure 8:**
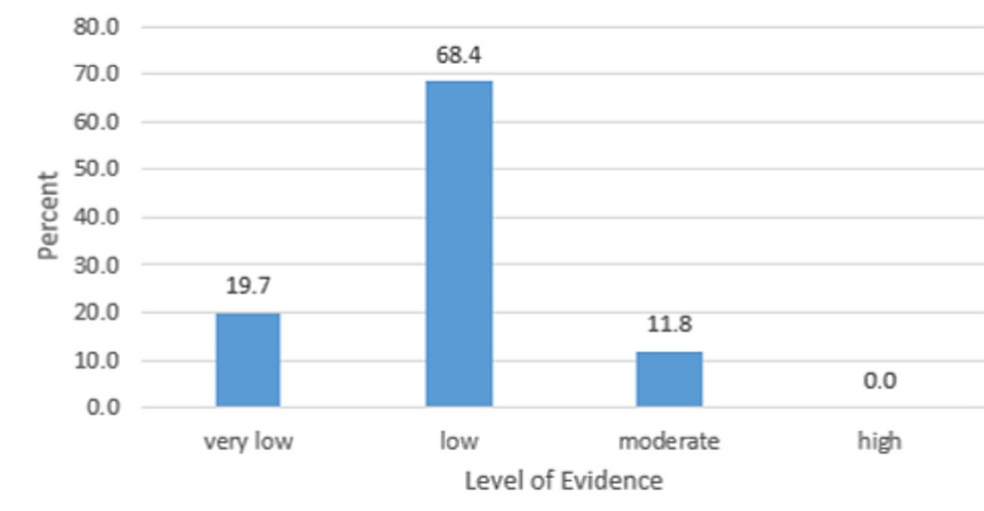
An evaluation of the top 100 most-cited articles by GRADE guidelines

Figure 8 displays an evaluation of the top 100 most-cited articles by GRADE guidelines. The top 100 most cited articles consisted of 34 review articles, conference statements, or consensus statements, 36 basic science articles, and 30 retrospective/prospective studies. After excluding review articles and conference statements, GRADE guidelines [4-6] (Table 5) showed that the level of evidence for the remaining articles was very low for 19.7%, low for 68.4%, moderate for 11.8%, and high for 0% of the articles (Figure 8).

**Table 5:** GRADE Guidelines

Study Design	Initial Quality of Evidence	Factors that Decrease the Quality Level	Factors that Increase the Quality Level
Randomized trials or double-upgraded observational studies	High	High likelihood of bias	Large effect
Downgraded randomized trials or observational studies	Moderate	Indirectness of evidence	All plausible confounding would reduce a demonstrated effect or suggest a spurious effect if no effect was observed
Double-downgraded randomized trials or observational studies	Low	Imprecision	Dose response gradient
Triple-downgraded randomized trials, downgraded observational studies, or case series/reports	Very Low	High probability of publication bias	

Discussion

Academic inquiries in the modern era are being pursued by a greater number of individual investigators than ever before, at around 7.8 million worldwide in 2013 [10]. A greater number of scientists correlates with a more considerable amount of scientific data. While an increased quantity of academic works leads to an overall increase in significant advancement in scientific understanding for the general public, it is worthwhile to analyze which articles seem to have risen above the rest. Such “foundational studies” are studies that have significantly impacted the course of academic progress in a particular field, resulting in frequent citation [11].

However, the argument can be made that an elevated number of overall citations may not be the best metric for determining an article’s influence. Instead, the best method may be to measure an article’s citations per year. If the paper continues to be cited over time, this demonstrates a continued impact on the scientific community. Bohl and Ponce 2017 found that when ranking articles based on citations per year as opposed to total citations, the citations that were in the top 100 were more recent publications that focused less on the understanding of the root cause or progression of a disease and more on surgical management and disease outcomes [12].

From our data of articles covering NF1 from 1963-2010, if all citations organize the articles, the premier publication is Neurofibromatosis: Conference Statement at 1527 citations since 1988 [7]. By this ranking, the second-most cited article is Wallace et al. 1990 at 1102 citations [13]. If the articles are organized by citations per year, Neurofibromatosis: Conference Statement is still the highest-ranked article at 47.7 citations per year, and Wallace et al.'s article from 1990 falls to rank five at 36.7 citations per year. The most recent publication within the top 10 within this ranking system is Williams et al. from 2009, with an average of 32.1 citations per year [14].

The Neurofibromatosis Conference Statement by Stumpf et al. in 1988 is referenced extensively throughout Neurofibromatosis literature as it is an early statement that set out to present information for the diagnosis of and management options for NF1, NF2, and NF variants [7]. Von Recklinghausen identified the disease in 1882, and over 100 years later, this panel consensus set forth the guidelines for clinical diagnosis of NF disorders based on physical characteristics [14-17]. Additionally, it discussed weak points in the current knowledge base, calling for academic research into topics that would serve as a springboard for future publications [7]. Some of the specific topics cited for future research include looking for possible genetic heterogeneity of NF patient families, isolating the NF genes to understand the pathophysiological progression of the disease, and acquiring prognostic data for survival and disease progression. One notable proposition that may have increased the amount this article was referenced is that the consensus panel calls for continued NF tumor DNA analysis. They state that NF2 tumor analysis had already shown potential for increasing understanding of all neoplasia, not simply neoplasia limited to NF [7].

Williams et al. 2009 is a review article that covers the history of NF1 disease presentation and makes recommendations for treatment based upon more recent findings [14]. The reason for its heightened citation count per year likely has to do with how recently the paper was published. A well-written review article summarizing the most relevant recent data about disease progression as well as the progress that has been made since foundational articles first laid forth the groundwork for treatment, is bound to be cited frequently within a certain duration of time. This is especially true immediately after publication, prior to new publications taking its place. Ultimately, guidelines will be updated as academic understanding progresses, and more review articles summarizing modern findings will be produced. As for specific findings of this review article, one notable statement was that there is interest in using small animals to study NF1 therapeutic methodologies. This is something that was briefly suspected as a future avenue for research in the NIH Conference Statement [7]. The article states that in combination with small animal imaging modalities, these models could allow researchers to more easily observe disease progression [14].

Our Lotka’s law analysis (Figure 6) found that the most prolific author within the top 100 most cited articles on NF1 was D.H. Gutmann, producing 13 articles within the top 100. Gutmann’s work often focused on defining the clinical basis for the management of NF1 and its individual characteristics. Some of his works include statements on peripheral nerve sheath tumors, optic gliomas, and cardiovascular manifestations of NF1, [18-20] genetic pathways integral to NF1 disease progression, [21,22] as well as an article identifying the NF1 gene product, NF1GRP [23]. His most cited work at 1017 citations is “The diagnostic evaluation of multidisciplinary management of neurofibromatosis 1 and neurofibromatosis 2”, an article that updated management guidelines based on recent evidence in a similar manner to that of the 1988 NIH Conference Statement before it, and the Williams 2009 review article after it [7,14,24]. Research that takes several bodies of work and synthesizes them into one cohesive statement for the most appropriate management of a disease is a springboard for future works, leading to multiple citations according to our findings.

Most articles in the top 100 are published within Cell (Figure 2). Notably, the year that had the most articles within the top 100 for NF1 was 1990 (Figure 1) with ten articles; six of which were from Cell. The cell had 11 articles within the top 100, meaning that these six articles may have had a significant influence not only on Cell’s high amount of top 100 NF1 articles but also 1990’s abundance of articles. It was in 1990 that the NF1 gene was cloned, and sections of its cDNA product were sequenced, resulting in each of these articles focusing on the NF1 gene and its GAP-related protein product [25-30]. These works likely had substantial collaboration, with researchers such as Viskochil, Cawthon, White, and Xu appearing in several publications. The cell is a journal focused on molecular biology, so, understandably, a year with an emphasis on discoveries related to the molecular pathway of NF1 would create a spike in publications of interest in Cell specifically. Besides an NF1 and NF2 update by Mulvihill et al., [31] the other publications in 1990 outside of Cell discussed the pathophysiology of malignant NF1 tumors [32,33] and added more information about the NF1 gene [13]. Of Gutmann’s 13 publications in the top 100, only his work on Cui et al. in 2008 was published in Cell [22].

Despite being highly cited, the majority of the articles found within this study held the GRADE score low. None of the studies in the top 100 most cited held the GRADE high. In the case of Neurofibromatosis 1, citation number is not a good surrogate for a quality paper according to the GRADE system [4-6]. One possible reason for this is the number of basic science articles (36) found within the top 100, which often fail to randomize and blind their studies, as well as a lack of randomized, controlled, and double- or triple-blinded studies.

Our analysis was not without limitations. Total citations and citations per year are not fool-proof methods for calculating publication impact. While total citations can often be skewed due to a more extended period of circulation, the inverse can be said with how our top article based on citations per year was still within its first and most relevant year. H-indices are not a perfect stand-in either, as the metric has potentially skewed results that can ignore the works of researchers who published several moderately successful publications or even a handful of outstanding articles [34]. Self-citations can additionally alter apparent citation counts when authors working together on multiple projects reciprocate references. While collaboration on top NF1 publications was noted, numerous studies of the effect of self-citation find that said citations usually account for less than 10% of citations and do not influence outcomes [35-37].

## Conclusions

In this study, we identified, characterized, and analyzed the top 100 most-cited works in NF1. This will serve as a historical point of reference for future research, a jumping point for those not familiar with the topic, and an educational foundation for future NF1 specialists and researchers. Citation count did not correlate with the quality of evidence. We suggest that this study be replicated every five years to assess the progress of NF1 research and to identify historical trends.
